# Accuracy and Precision of Three-Dimensional Low Dose CT Compared to Standard RSA in Acetabular Cups: An Experimental Study

**DOI:** 10.1155/2016/5909741

**Published:** 2016-07-10

**Authors:** Cyrus Brodén, Henrik Olivecrona, Gerald Q. Maguire, Marilyn E. Noz, Michael P. Zeleznik, Olof Sköldenberg

**Affiliations:** ^1^Department of Molecular Medicine and Surgery, Karolinska Institutet, 17176 Stockholm, Sweden; ^2^School of Information and Communication Technology, KTH Royal Institute of Technology, 16440 Stockholm, Sweden; ^3^Department of Radiology, New York University School of Medicine, New York, NY 10016, USA; ^4^School of Computing, College of Engineering, University of Utah, Salt Lake City, UT 84109, USA; ^5^Department of Clinical Sciences, Danderyd Hospital, Karolinska Institutet, 18288 Stockholm, Sweden

## Abstract

*Background and Purpose*. The gold standard for detection of implant wear and migration is currently radiostereometry (RSA). The purpose of this study is to compare a three-dimensional computed tomography technique (3D CT) to standard RSA as an alternative technique for measuring migration of acetabular cups in total hip arthroplasty.* Materials and Methods*. With tantalum beads, we marked one cemented and one uncemented cup and mounted these on a similarly marked pelvic model. A comparison was made between 3D CT and standard RSA for measuring migration. Twelve repeated stereoradiographs and CT scans with double examinations in each position and gradual migration of the implants were made. Precision and accuracy of the 3D CT were calculated.* Results*. The accuracy of the 3D CT ranged between 0.07 and 0.32 mm for translations and 0.21 and 0.82° for rotation. The precision ranged between 0.01 and 0.09 mm for translations and 0.06 and 0.29° for rotations, respectively. For standard RSA, the precision ranged between 0.04 and 0.09 mm for translations and 0.08 and 0.32° for rotations, respectively. There was no significant difference in precision between 3D CT and standard RSA. The effective radiation dose of the 3D CT method, comparable to RSA, was estimated to be 0.33 mSv.* Interpretation.* Low dose 3D CT is a comparable method to standard RSA in an experimental setting.

## 1. Introduction

Radiostereometric Analysis (RSA) is the gold standard for precise monitoring of micromovements of orthopaedic joint implants [1–4]. However, since not every hospital does have the stereoradiographic facilities needed for doing standard RSA imaging, it is therefore important to consider alternative techniques to follow prosthesis migration over time.

Current computed tomography (CT) scanners can routinely provide high-resolution volume data with voxels of submillimeter size in all dimensions. Therefore, the potential exists for detecting the small tantalum beads implanted as RSA markers in CT volumes with reasonable accuracy, and then this data can be used to calculate the marker positions.

At the Karolinska Institute a three-dimensional (3D) CT technique (3D CT) has been developed that could potentially detect migration and subsequent osteolysis in hip prostheses [5, 6]. The aim of this study was to validate this low dose 3D CT as a tool for migration assessment of acetabular components in total hip arthroplasty (THA).

## 2. Materials and Methods

### 2.1. Experimental Setup

**Figure 1:**
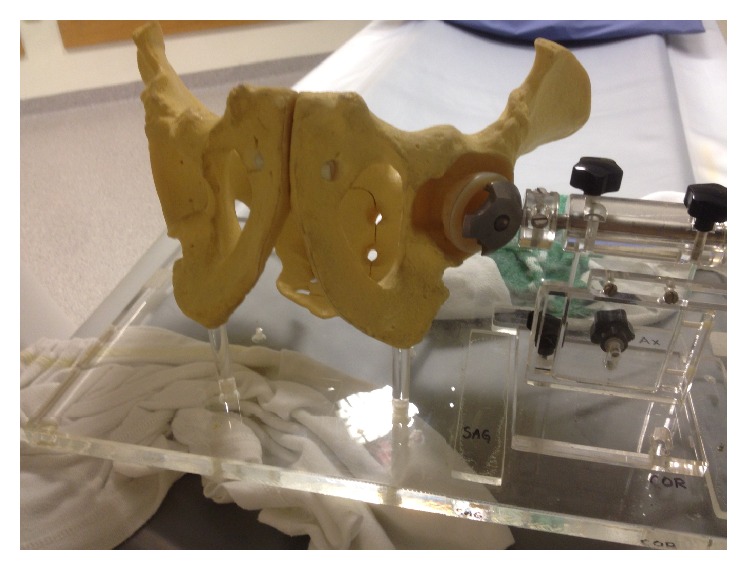
Pelvic model with jig holding the cemented cup in place for the examinations. The jig allows precise translations in *x*-, *y*-, and *z*-axis by 1.0 mm increments and rotations in 1.0° increments about the *x*-axis.

A plastic model of the human pelvis (Sawbones, Vashon, WA, USA) was marked with nine 1.0 mm tantalum beads. To simulate a typical marker configuration in THA, the markers were placed in the periacetabular bone using the same procedure as when marking a patient during surgery. We used two implants mounted consecutively to the pelvic model: a cemented (Müller Exceed*™*, Biomet, Warsaw, Indiana, USA) and an uncemented (T.O.P.®, Waldemar LINK GmbH & Co. KG, Hamburg, Germany) acetabular cup. They were implanted in the model after being marked with tantalum beads in a circular fashion in the periphery of the opening of the polyethylene liner. The cups were held by a jig that allowed translations in *x*-, *y*-, and *z*-axis by 1.0 mm increments. Each cup could also be rotated in 1.0° increments about the *x*-axis as shown in Figure 1. Six positions for each cup relative to the pelvic model were chosen. For each position we added translation and/or rotation to the cup to simulate a movement of the component relative to the pelvic model. For each position a double examination of RSA and a double examination of CT were conducted. Within each scan in a double examination the model was moved without changing the position of the cup relative to the pelvic model in order to simulate movement of a patient between the two examinations.

The following procedure was performed to measure the migration of the implant in relation to the model: (1) the pelvic phantom including the jig was placed in the RSA calibration cage at the point of intersection of the central radiograms above the RSA calibration cage, (2) one set of radiographs was taken (position 1_RSA_, series 1_RSA_), (3) the calibration cage, the X-ray tubes, and the phantom were repositioned (without moving any of the phantom's components), (4) one set of radiographs was taken (position 1_RSA_, series 2_RSA_), (5) the model was moved (without moving any of the phantom's components) to the CT table, (6) one CT scan was done (position 1_CT_, series 1_CT_), (7) the phantom was repositioned (without moving the cup in relation to the pelvis) in the CT scanner, (8) one CT scan was done (position 1_CT_, series 2_CT_), and (9) the prosthesis was moved 1.0 mm in relation to the pelvis, along the *x*- or *y*-axis and rotated 1.0° around the *x*-axis to simulate migration of the implant. This resulted in all migrations being along the *x*-, *y*-, and *z*-axes for translations and about the *x*-, *y*-, and *z*-axes for rotations. Steps (1) to (9) were repeated a total of 6 times for each cup, giving us, for each RSA and CT examination, position 1, series 1 and 2; position 2, series 1 and 2; and so on.

### 2.2. The RSA Method

Uniplanar calibration cage (Cage 43; RSA Biomedical AB, Umea, Sweden) was used. Digital radiographs (Bucky Diagnostic; Philips, Eindhoven, the Netherlands) were then taken using 2 X-ray sources angled at 40° to each other. The exposure was set to 125 kV and 2.5 mAs for each X-ray tube. The measurement and migration analyses were done with the UmRSA 6.0 computer software (RSA Biomedical, Umea, Sweden).

The stereoradiographs that composed the RSA examinations were conducted by an experienced radiologic nurse and a physician with extensive experience using RSA did the analysis. The condition number and the mean error were calculated. The condition number assesses the distribution of markers and should be below 100–110 to be reliable [4]. The mean error represents the stability of the markers and is acceptable if it is under 0.35 mm [7, 8]. In our study, all condition numbers were below 100 and all mean errors below 0.30 mm.

### 2.3. The 3D CT Method

For each position, two CT scans were obtained. All volumes were acquired using the same scout view. A clinical CT scanner (Discovery CT750HD, GE Healthcare, USA) was used. Images were acquired at 120 kVp, 10 mAs. The scanner software did not permit reducing the mAs more. Volumes were reconstructed with an in-plane resolution of 0.6*∗*0.6 mm at 0.3 mm increments.

### 2.4. Image Analysis

**Figure 2:**
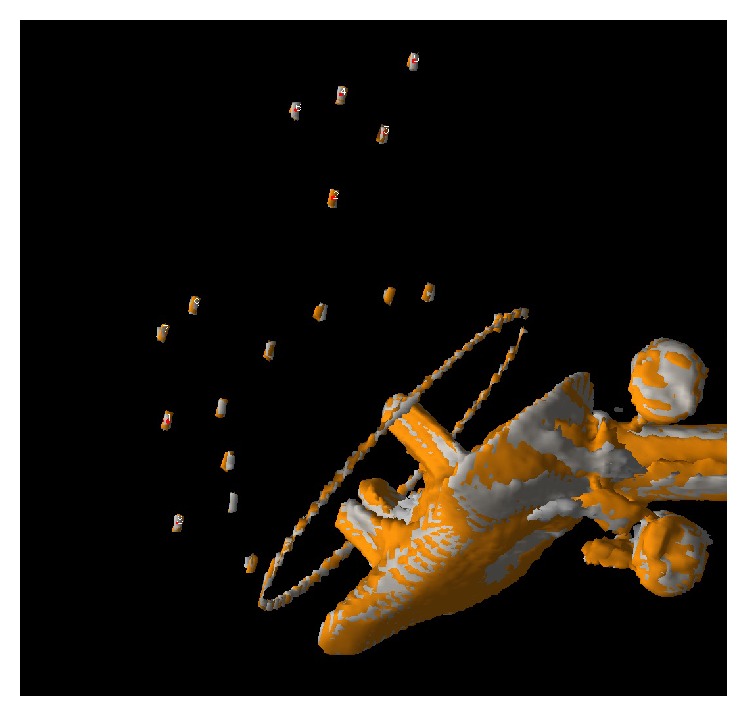
Volume fusion of the transformed and reference volume. In this experiment the relative movement of the cup is zero. The overlapping pattern between the two examinations indicates that the surface representations are closer than the smallest voxel elements and that visually the precision of this method is good.

In order to easily compare two CT volumes, it is helpful if they are viewed simultaneously in the same spatial alignment. For this we used a 3D volume image processing tool which includes functions for volume registration, fusion, and data analysis [5, 6]. This registration algorithm has been described previously and extensively validated [9–11]. The semiautomated image registration procedure has a graphical interface and is used to perform landmark-based fusion of two volumes. In first step, one of the two volumes of a pair was spatially aligned with the second volume (the reference volume). The spatially aligned volume is called the transformed volume. This transformation is possible by using landmarks chosen manually on the cohomologous tantalum beads in the pelvis of the two volumes as shown in Figure 2 [5, 6]. The program used these manually designated preliminary landmarks as starting points for an automated process that calculated the best fit center of the tantalum beads and designated the final landmarks at the centroid of each bead before the transformation. The transformed volume could then be compared to the reference volume. In a second step, we utilize the markers in both cups of the paired volumes (as landmarks) in order to perform a numerical analysis to calculate precision and accuracy. All the measurements were done by a physician experienced in the CT method who was different from person who did the RSA analysis.

### 2.5. Precision

The precision of a measurement is “the degree to which repeated measurements under unchanged conditions show the same results” [12, 13]. Precision was calculated as the difference between the double measurements (series 1 and 2) at one predetermined position of the cup relative to the pelvic model. For instance, for *x*-translation (*xt*), *d*_prec_*xt*__ = *xt*_*p*_1:1__ − *xt*_*p*_1:2__, where *d*_prec_*xt*__ is the difference between position 1 series 1 (*p*_1:1_) and position 1 series 2 (*p*_1:2_) [13]. If the precision of the modality were perfect, then the difference between these two positions would be null. When viewing the two images, this would look like a perfect fusion between the two implants, since no movement between the implants relative to the pelvic model occurred between these scans (Figure 2).

For the precision measurements, we used as many tantalum beads as could be visualized in each modality. In the 3D CT mode we used nine markers for bone and nine markers for the prosthesis for the cemented cup. For the uncemented cup, we used nine markers for bone and twelve for the prosthesis. For the standard RSA we used six markers for the cemented and four markers for the uncemented cup. This reduced number of markers was due to the fact that the standard RSA method suffered from marker occlusion, even in our experimental setting.

### 2.6. Accuracy

The definition of accuracy is “the degree of closeness between a measured value and the true value and contains both random and systemic errors” [12, 13]. The accuracy of standard RSA was, in this laboratory setting, assumed to be perfect; that is, standard RSA measures the true migration of the implant [14]. The standard RSA measurements were therefore used as the gold standard measurements when we calculated the accuracy of the 3D CT method. For assessing accuracy we had to use only the tantalum beads that could be visualized in both modalities. We used six tantalum beads with the cemented cup and four with the uncemented cup and seven markers for bone with both cups. In a first step, migration was calculated pairwise in positions 1-2, 3-4, and 5-6 to get independent measurements, in both RSA and CT.

*In RSA*. For instance, for *x*-translation, RSA_*xt*_1-2__ = *xt*_*p*_1__ − *xt*_*p*_2__, where RSA_*xt*_1-2__ is the migration of the prosthesis from positions 1 (*p*_1_) and 2 (*p*_2_)

*In CT*. Consider the following: CT_*xt*_1-2__ = *xt*_*p*_1__ − *xt*_*p*_2__, where CT_*xt*_1-2__ is the migration of the cup from positions 1 (*p*_1_) and 2 (*p*_2_).

The difference between the migration values of the two modalities should ideally be zero if the accuracy is perfect; that is, *d*_accurCT_*xt*__ = RSA_*xt*_1-2__ − CT_*xt*_1-2__ = 0, where *d*_accurCT_*xt*__ is the accuracy for the *x*-translation.

### 2.7. Radiation

The CT effective dose was calculated using the manufacturer stated dose length product (DLP mGy-cm) and combining this with the normalized effective dose DLP conversion factor (*k* mSV/(mGy-cm)) for the human pelvis [15]. The RSA effective dose was estimated with a Monte Carlo simulation using a software program xDose in our hospital developed by the National Radiological Protection Board [16].

### 2.8. Statistics

We calculated precision and accuracy for translations and rotations in the *x*-, *y*-, and *z*-axes. The data was first examined to determine if it followed a normal (Gaussian) distribution by histograms, box, density, and quantile-quantile plots so that the standard deviation (SD) could be used. We calculated the precision for standard RSA and 3D CT as 2.45*∗*SD (6 degrees of freedom (d.o.f.)) of the difference between the double examinations (*d*_prec_). The 95% quantile for the *t*-distribution with 6 d.o.f. is 2.45, and this was chosen for precision since only random errors are included in precision measurements. We calculated the accuracy for 3D CT using the root mean square error (RMS) as 2.57*∗*RMS (5 d.o.f.). This gives a measure of the magnitude of a varying quantity and was chosen since the difference between the standard RSA and the 3D CT method could be both positive and negative. The 95% quantile for the *t*-distribution with 5 d.o.f. is 2.57 and was chosen because accuracy involves both systemic and random errors. SPSS 22 for Mac was used for all statistical calculations.

## 3. Results

The precision for 3D CT was comparable to standard RSA, ranging between 0.01 and 0.09 mm for translations and 0.06 and 0.29° for rotations. For standard RSA, the precision ranged from 0.04 to 0.09 mm for translations and 0.08 to 0.32° for rotations, respectively (Table 1). All markers (twelve for the uncemented and nine for the cemented cup) could be used for 3D CT whereas six and four markers were used for standard RSA for the cemented and uncemented cups, respectively.

The accuracy ranged from 0.07 to 0.32 mm for translations and 0.21 to 0.82° for rotation for the 3D CT method (Table 2). The measurements for the uncemented cup had lower accuracy. This was explained by the fact that the same rigid body model was used when comparing the two methods and via standard RSA we could only identify 4 (out of 9) markers.

The landmark designation procedure in the 3D CT was rapid and required less than five minutes per volume. Since the 3D volumes could be freely rotated and viewed from arbitrary angles, it was easy to differentiate between tantalum beads. In contrast, in standard RSA tantalum beads were harder to identify than in 3D CT. The effective radiation dose was estimated to be on an average 0.33 mSv per scan for the 3D CT method and 0.1 mSv for standard RSA.

## 4. Discussion

To our knowledge, this is the first study comparing a 3D CT and a RSA method. There have however been numerous publications based on our CT method used for other applications such as acetabular loosening, motion analysis of disc replacements, and cup wear [5, 6, 17]. Numerous publications on RSA have shown that it can be used as an early detector of migration of an implant, which is an early sign for risk of revision [18, 19]. RSA has therefore been suggested to play a role in evidence based introduction of new implants. For knee implants, migration of the tibia component of more than 1.6 mm during the first year is unacceptable and indicates a high risk for revision [19]. For hip stems the prediction of failure of implants is due to the shape of the implant. Migration exceeding 0.85 mm within the first six months is a predictor for implant failure for anatomical cemented stems [20].

Our aim in this phantom study using a pelvic model was to compare our new 3D CT method with standard RSA in terms of precision and accuracy in order to see if this new method could be used to detect early signs of loosening of a prosthetic implant in patients who also have implanted markers. It could therefore provide an alternative for evidence based introduction of new implants. We found comparable precision for 3D CT compared to standard RSA in acetabular components for THA. In standard RSA, even in our experimental setting, both our cups, but especially the uncemented cup, suffered from marker occlusion, that is, not being able to use all tantalum beads. This problem is common in RSA studies, especially around uncemented acetabular components; hence other RSA alternatives have been developed to solve this problem such as model based RSA [21–23].

We also found a high degree of accuracy for the 3D CT method when using standard RSA as the gold standard. In the uncemented cup we could only use four tantalum beads that could be visualized on both images in standard RSA and thus only these markers could be used when comparing migration between methods. These markers did not form an optimal stable rigid body for 3D CT; therefore the results of the accuracy of the uncemented cup were not as satisfying as those for the cemented cup.

In this model study, the radiation dose was comparable to RSA. The radiation dose in CT is highly dependent on the machine and the protocol used for the examination. In this case, the average effective dose was estimated to be 0.33 mSv. This is a low dose and can be compared to the RSA effective dose estimated to be 0.1 mSv in this study.

CT is easily acquired and the examination can be performed on any modern CT scanner. The acquisition is fast, and patient positioning in the scanner is not vital, since the volume can be transformed into an arbitrary spatial orientation for viewing and processing.

There are several potential benefits from using CT as opposed to RSA. It greatly speeds up the marking process, since marker identification becomes trivial when utilizing powerful, interactive 2D and 3D visualization tools applied to the CT volume data. This enables 3D evaluation of marker configuration and distribution. In addition to reporting the relative motion numerically, the CT method gives immediate visual feedback in both 2D and 3D, with volumes displayed either side by side or fused. The quality of the registration, in this case based on the markers attached to the bone, as well as the relative movement, could be visually evaluated. Any point in these volumes can be accessed and designated, so there is potential for studying the relative movement at any location. Another advantage is that landmarks can be added on structures other than tantalum beads, if the stability of the rigid body is not sufficient [24]. Another advantage of 3D CT could be the simultaneous evaluation of, for example, osteolysis in clinical cases which has not been evaluated in this experimental study.

Disadvantages of the proposed method are that it is new and relatively untested for this application and has not been validated as much as RSA. Additionally, it requires user interaction which could vary from one operator to another. To our knowledge, there is no commercially available 3D CT software at present.

The limitation of our study was that we did not use an instrument that could directly measure migration of the implant for comparison with the 3D CT method. Instead we used the RSA method as a gold standard that has a documented small error in precision and accuracy [4].

## 5. Conclusion

In conclusion, 3D CT has comparable precision to standard RSA and an acceptable accuracy. This CT method could potentially be used to evaluate patients with RSA markers, thus avoiding the inability to evaluate these patients over time due to the lack of facilities for doing stereoradiographs. Further, the effective dose associated with CT is becoming comparable to that of two planar X-rays. However, further clinical studies with the 3D CT method on patients are necessary before it can be used as an alternative method to RSA.

## Figures and Tables

**Table 1:** Precision of 3D CT compared to standard RSA.

	Standard RSA				3D CT			
	2.45 *∗* SD	Mean	Min	Max	2.45 *∗* SD	Mean	Min	Max
Uncemented cup								
Translation (mm)								
*x*	0.07	0.00	−0.03	0.05	0.01	0.00	0.00	0.01
*y*	0.05	0.02	−0.02	0.04	0.04	−0.01	−0.04	0.00
*z*	0.09	0.03	−0.02	0.09	0.09	−0.01	−0.09	0.01
Rotation (°)								
*x*	0.22	−0.05	−0.15	0.12	0.10	−0.01	−0.10	0.01
*y*	0.13	0.00	−0.10	0.05	0.06	−0.02	−0.05	0.00
*z*	0.08	0.00	−0.03	0.05	0.21	−0.07	−0.21	0.01
Cemented cup								
Translation (mm)								
*x*	0.08	0.00	−0.04	0.05	0.04	−0.01	−0.03	0.00
*y*	0.04	0.00	−0.02	0.02	0.04	−0.01	−0.04	0.00
*z*	0.09	0.00	−0.05	0.05	0.06	−0.02	−0.06	0.00
Rotation (°)								
*x*	0.19	−0.03	−0.16	0.06	0.14	−0.02	−0.11	0.02
*y*	0.32	0.02	−0.17	0.14	0.29	−0.14	−0.24	0.02
*z*	0.12	0.02	−0.03	0.10	0.27	−0.05	−0.27	0.02

**Table 2:** Accuracy for 3D CT.

	2.57 *∗* RMS	Mean	Min	Max
Uncemented cup				
Translation (mm)				
*x*	0.29	0.05	−0.11	0.19
*y*	0.28	−0.06	−0.13	0.05
*z*	0.32	0.04	−0.06	0.26
Rotation (d)				
*x*	0.82	−0.15	−0.56	0.17
*y*	0.71	−0.18	−0.52	0.06
*z*	0.43	−0.04	−0.22	0.10
Cemented cup				
Translation (mm)				
*x*	0.19	0.01	−0.17	0.17
*y*	0.07	0.01	−0.08	0.07
*z*	0.08	0.00	−0.10	0.07
Rotation (d)				
*x*	0.21	0.01	−0.15	0.28
*y*	0.44	0.05	−0.43	0.49
*z*	0.26	−0.01	−0.33	0.22
